# Inflammatory mechanisms contributing to retinal alterations in HIV infection and long-term ART

**DOI:** 10.4102/sajhivmed.v25i1.1548

**Published:** 2024-03-26

**Authors:** Lungile M. Buthelezi, Alvin J. Munsamy, Khathutshelo P. Mashige

**Affiliations:** 1 Department of Optometry, School of Health Sciences, University of KwaZulu-Natal, Durban, South Africa

**Keywords:** retina, blood-retina barrier, HIV, antiretroviral therapy, inflammation, optical coherence tomography

## Abstract

People living with HIV (PLWH) may face an increased risk of eye complications associated with ageing, chronic inflammation, and the toxicity arising from long-term antiretroviral therapy (ART). This review aims to understand how inflammatory pathways contribute to retinal alterations observed in PLWH on long-term ART. This review was conducted using four electronic database searches, namely Scopus, Hinari, Google Scholar, and PubMed; from 1996 (when ART became available) until January 2022, without language restriction. Sources from clinical trials, meta-analyses, randomised controlled trials, and systematic reviews were used. Dysregulated para-inflammation (chronic inflammation) damages the blood-retina barrier, resulting in the altered retinal immune privilege and leading to the development of retinal and blood vessel changes. There is an interplay between the effects of the disease versus ART. ART causes mitochondrial toxicity, which affects the retinal ganglion cells and retinal pigment epithelium (RPE) due to oxidative stress. Infection by HIV also affects retinal microglia, which contributes to RPE damage. Both of these mechanisms affect the blood vessels. Assessing the integrity of the inner and outer blood-retina barrier is a pivotal point in pinpointing the pathogenesis of inner retinal alterations. Optical coherence tomography is a valuable tool to assess these changes. There is a paucity of research to understand how these structural changes may affect visual function, such as contrast sensitivity and colour vision.

**What this study adds:** This review identifies knowledge gaps affecting retinal research and HIV patient care. It suggests that the compromised blood-retina barrier could be the entry point for HIV and ART-induced inflammation, providing insight on previously uncharted mechanisms. This leads to efficient diagnosis and management thus preserving visual function and quality of life.

## Introduction

**FIGURE 1:**
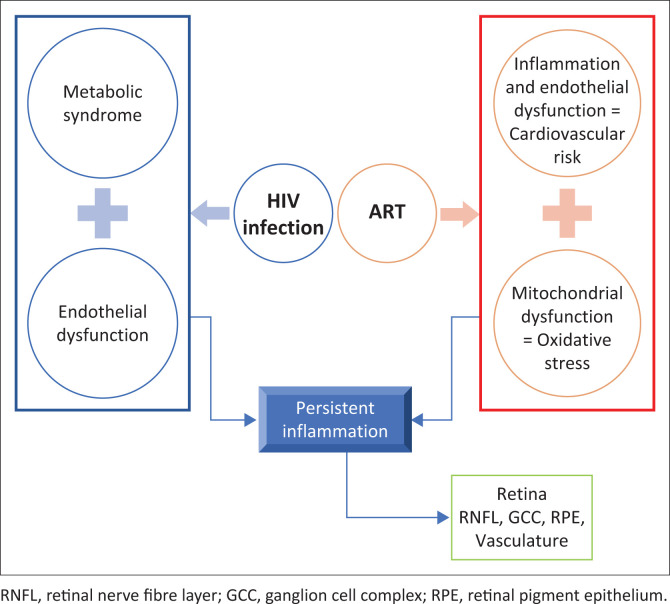
Illustration of the effects of HIV infection and long-term ART use on the body and the retina.

Since the introduction of antiretroviral therapy (ART), opportunistic infections and HIV-related retinal diseases have lessened. The advantages of ART are the restoration of pathogen-specific immune responses, facilitated by inhibited HIV replication and increased CD4 cell count.^1^ Although ART has been shown to improve life expectancy and health, it has some side effects. People living with HIV (PLWH) receive lifelong ART. However, the long-term effects in the eyes of PLWH are largely unknown. Figure 1 shows a simplified illustration of the effects of HIV infection and long-term ART use on the body and how the eye (retina) may be affected. HIV infection causes metabolic and endothelial dysfunction. The use of long-term ART perpetuates endothelial dysfunction which results in an increased risk of cardiovascular disease (CVD). Furthermore, ART causes mitochondrial dysfunction which contributes to oxidative stress. Both these pathways (endothelial and metabolic dysfunctions) result in persistent inflammation which may disrupt retinal functions and manifest as alterations in retinal thickness and vasculature damage.

Most drug toxicities can be categorised into four types based on the source of their underlying mechanism: (1) hypersensitivity and related immunological reactions; (2) off-target pharmacology; (3) biological activation to toxic metabolites; and (4) idiosyncratic toxicities.^2^ Immunological reactions are seen in immune recovery following ART initiation, which may lead to the occurrence of ocular conditions such as immune recovery uveitis, especially among individuals with low CD4 count at ART commencement.^3^ The continual immune activation, sub-optimal HIV containment and incomplete immune restoration may predispose patients to various ocular conditions. Those who are on long-term ART have demonstrated abnormal cellular functions where ART has been shown to cause low-grade inflammation, mitochondrial dysfunction, and senescence (biological ageing) even in the early stages of drug intake.^4^

There is a need to identify and validate biomarkers for predicting adverse reactions and improving current toxicological models. In eye care, spectral-domain optical coherence tomography (SD-OCT) technology has gained prominence as the gold standard for retinal imaging in various eye diseases. Although OCT technologies have been widely adopted in clinical practice, their utilisation within the context of HIV-related retinal management is not well established. This review appraises the literature on the underlying aspects of HIV/ART-induced ocular changes, highlighting some pathophysiological features of this disease along with the role played by HIV itself and the interactive activities of some antiretroviral drugs. Furthermore, the usefulness of SD-OCT for assessing the integrity and stability of retinal structures will be investigated. Studies for this literature search were retrieved via a systematic web-based database search. Five electronic databases – Google Scholar, Embase, Hinari, MEDLINE, and PubMed – were searched from 1996 (when ART became available) until January 2022, without language restriction. Studies published before 1996 were excluded. Literature was then categorised according to their different *foci* on issues related to the purpose of the study.

## Current characterisation of the retina in HIV infection

### HIV factors as predictors of retinal morphological changes

HIV retinopathy occurs primarily in patients with CD4 counts below 50 cells/mm^3^. Most studies in Table 1 have categorised patients according to their nadir CD4 count, in particular below and above a threshold of 100 cells/mm^3^. This suggests that patients with higher (> 100 cells/mm^3^) nadir CD4 counts are unlikely to manifest retinal pathology. However, recent studies have used 350 cells/mm^3^ as a threshold for immunocompetence.^14,15,16^

**TABLE 1:** Studies (cross-sectional) reporting on the associations between HIV characteristics and retinal alterations.

Study	*N* (eyes)	HIV factors	Time since diagnosis (months)	Duration of treatment (months)	Mean nadir CD4 count (cells/µL)	Mean CD4 count (cells/mm^3^)	Viral load (copies/mL)	Retina associations
Plummer et al.^5^	28	NR	NR	NR	NR	NR	NR	RNFL thickness: HIV+: 0.23; HIV–: 0.28 (*P* = 0.011). Thinner mRNFL in HIV+. HIV-positive patients have changes in the optic disk and RNFL as compared with HIV– controls.
Kozak et al.^6^	65†	CD4 > 100 cells/mm^3^	NR	NR	NR	NR	NR	Thinner mRNFL: CD4+ > 100 (103.30 um ± 9.28 µm); CD+ < 100 (90.10 µm ± 12.50 um) Difference between low CD4 HIV-positive and high CD4 HIV-positive/HIV-negative groups was most evident in sup and inf retinal areas.
Besada et al.^7^	13‡	CD4 > 100 cells/mm^3^	NR	NR	NR	NR	NR	No correlation between CD4 count, VL, years since diagnosis of HIV with effects on the retina (ppRNFL).
Faria et al.^8^	73	CD4 > 100 cells/mm^3^	85.88 ± 10.03	76.96 ± 10.34	301.08 ± 31.11	502.29 ± 41.36	NR	Average ppRNFL thickness is significantly thinner in lower CD4 counts (*P* = 0.002).
Demirkaya et al.^9^	92	Immune competent	NR	12.00	180.00	595.00	Undetectable	Increased total peripheral retinal thickness (+4.6 µm, *P* = 0.029). Increase in INL (+1.04 µm, *P* = 0.006), increase in OPL (+0.95 µm, *P* = 0.006) thickness. Central outer segment layer thickness was negatively associated with ART duration (− 0.141 µm/year; *P*-value: 0.048).
Pathai et al.^10^	242	ART treatment	NR	58.00	127.00 (76–171)	468.00	84.3% of group undetectable	Arteriolar diameters narrowed with increasing duration of ART (independent of age (167.83 µm < 3 years of ART vs. 158.89 µm > 6 years) (*P* = 0.02). Retinal arteriolar diameters narrowed with VL > 10 000 copies/mL while on ART (*P* = 0.05).
Pathai et al.^11^	225	ART treatment	NR	56.50 (34–74)	136.00 (77–175)	468.00 (327–607)	15.1% of group with detectable load	Longer duration of ART was associated with a thinner RNFL. No association was detected with type of ART.
Arcinue et al.^12^	10	ART treatment	NR	NR	NR	NR	NR	HIV group had significantly less mean cone photoreceptor density compared with controls. HIV-positive group had a thicker average retinal thickness at the foveal centre. There was thicker inner retina in the HIV-positive group’s eyes.
Cetin et al.^13^	48	NR	21.90 ± 29.80 (0–120)	16.30 ± 23.20 (0–100)	NR	426.00 ± 226.00	NR	IPL, sup PR (ETDRS 3 mm was thinner) (*P* = 0.033). Sup- and Nas- RPE layers were thinner (*P* < 0.05).
Munsamy et al.^14^	30	CD4 > 350 cells/mm^3^	NR	72.00 (48–96)	NR	594.00 (460–771)	0.00 (0–46.25)	Thicker mean global temp subfields at ETDRS 3 mm (*P* = 0.047), ETDRS 6 mm (*P* = 0.03), and mean global temp subfield volume at ETDRS 3 mm was increased (*P* = 0.02). Significant association with the odds of having thicker layers and ppRNFL (OR 1.11) with higher CD4 percentage.

*Source*: Please see the full reference list of the article for more information

NR, not reported; ART, antiretroviral therapy; sup, superior; inf, inferior; nas, nasal; temp, temporal; RNFL, retinal nerve fibre layer; mRNFL, macula retinal nerve fibre layer; ppRNFL, peripapillary retinal nerve fibre layer; VL, viral load; OPL, outer plexiform layer; IPL, inner plexiform layer; PR, photoreceptor; INL, inner nuclear layer; RPE, retinal pigment epithelium; ETDRS, Early Treatment Diabetic Retinopathy Study; OR, odds ratio.

† , Number of eyes = 113;

‡ , Number of eyes = 26.

There is agreement among numerous researchers that there is peripapillary retinal nerve fibre layer (ppRNFL) loss in PLWH without retinitis in patients with low CD4 counts.^5,6,7,8^ However, the opposite is found in patients with higher CD4 counts, especially newly diagnosed patients compared to those on ART.^6,9,13,14^ Although viral load (VL) was not reported in some studies, we postulate that higher viraemia damages the optic nerve, and recovery of the immune system may restore the morphological structure, which is later affected by chronic low-grade inflammation possibly caused by both the disease and ART. This points to the hypothesis of optic nerve cell plasticity, where the ppRNFL has an adaptive response to its environment in response to various factors.

Similar reports are found about macula thickness. The same studies consistently show that PLWH with low CD4 counts exhibit a reduction in retinal thickness, particularly in the macula region and specific retinal layers (RNFL, inner plexiform layer [IPL], RPE). The severity of immune compromise appears to correlate with the extent of retinal thinning. Individuals with CD4 counts < 100 cells/mm³ have a decrease in retinal thickness. However, the opposite is found in immune-competent states (approximate CD4 > 200 cells/mm^3^). Overall, the studies consistently demonstrated a significant association between higher CD4 counts, and retinal thickening (overall macula thickness, macula retinal nerve fibre layer [mRNFL], and inner nuclear layer [INL]).^6,8,9,12,14^ This may be due to a gradual improvement in retinal thickness over time following ART initiation and subsequent immune reconstitution; further supporting the theory of retinal plasticity. The increased thickening may also reflect low-grade inflammatory activity causing increased vascular permeability (through disruption of the blood-retinal barrier), and pro-inflammatory cytokines and chemokines (which promote the proliferation and activation of retinal glial cells resulting in increased production of extracellular matrix components).

## Mechanisms of retinal pathology – Disease, drug, or both?

The pathogenesis of HIV- or ART-related complications remains uncertain and speculative. Therefore, it is a challenge to differentiate retinal abnormalities that are related to the drug, and those that are related to the virus itself, except when obvious adverse effects or mechanisms have been linked to individual drugs. Nonetheless, any retinal changes that occur are attributable to an interplay of both the disease and drug factors.

### Mechanisms of ART-induced retinal toxicity

#### Cellular neuro-inflammatory stress

We found no studies that directly investigated the possible effects of ART on the retina in PLWH. An earlier review by Abers et al.^17^ found that zidovudine, abacavir, efavirenz, didanosine, stavudine, and ritonavir were responsible for mitochondrial dysfunction which manifested as damage to the RPE and with macula telangiectasias and intraretinal crystalline deposits. A case report by Pereira et al.^18^ also found atrophy of macula RPE (Bull’s eye maculopathy) in a patient on efavirenz for 10 months. Although they did not report on the mechanism of toxicity, they postulate that an increased plasma concentration of the drug may cause retinal changes. Similar reasoning of accumulation of a xenobiotic substance in optic nerve cells was reported by Riva et al.,^19^ who found optic neuropathy in a patient on a combination elvitegravir/cobicistat/emtricitabine/tenofovir alafenamide. Other studies report that ritonavir causes bilateral maculopathy with RPE atrophy.^20,21,22^ All reports hypothesise that ritonavir may lead to hyperplasia of the RPE and subsequent retinal degeneration. Although further studies are required to characterise ART toxicity, we propose that most of these drugs affect the RPE through different mechanisms of inflammation.

Cellular pro-inflammatory stress is the:

[*T*]ypical morphofunctional changes in cells in response to damage or threat of damage, aimed at adaptation of the cell, tissue/organ, and organism to the action of damaging factors of various nature.^23^

Cellular stress is comprised of various processes that are additive, such as oxidative stress, cell response to DNA damage, mitochondrial stress, formation of an intracellular network of signalling pathways of cellular stress, and formation of pro-inflammatory receptor and secretory cell phenotype.^24^ Prolonged exposure to nucleoside/nucleotide reverse transcriptase inhibitors (NRTIs) inhibits DNA polymerase gamma which functions in mitochondrial DNA (mtDNA) replication and maintenance.^17^ This results in decreased mtDNA and oxidative stress,^25^ leading to possible neuronal death.^26^ Oxidative stress is attributed to RPE damage and outer retinal layer dysfunction.^27^ Consequently, when these structures are damaged, they may favour the progression of retinal dystrophy typically seen as macula morphological alterations.^28^ Riva et al.^19^ reported a case of combination elvitegravir/cobicistat/tenofovir/emtricitabine with no other comorbidities causing toxic optic neuropathy due to an accumulation of a xenobiotic substance in optic nerve cells affecting the mitochondria of retinal ganglion cells (RGCs) and the papillomacula bundle.

The administration of NRTIs results in inflammatory damage to sensory axons and dorsal root ganglia.^29^ Small, unmyelinated fibres are vulnerable to these effects of NRTIs. In the eye, this may impact the unmyelinated axons of RGCs that form the RNFL. Didanosine is associated with retinopathy caused by RPE atrophy, resulting in mottling that presents as circumscribed lesions at the periphery of the fundus.^30^ Abers et al.^17^ suggest an eye examination every 6 months to 12 months to monitor for didanosine-induced retinal toxicity. Roe et al.^31^ also reported ritonavir intake for more than 12 months can result in RPE damage but with macular telangiectasias and intraretinal crystalline deposits.^31^

#### Para-inflammatory response resulting in secondary mitochondrial dysfunction

Neuroinflammation describes immune-driven pathology which occurs in the course of disease or infection in the brain tissue.^32^ This tissue state can be identified by four hallmarks: (1) high levels of pro-inflammatory cytokines; (2) microglia activation; (3) infiltration of peripheral leukocytes (e.g. bone-derived monocytes, T-cells); and (4) blood-brain barrier breakdown and neuron death. During the course of the pathology, the neuro-immune system may experience some homeostatic challenges which lead to para-inflammation.^32^ Para-inflammation is a tissue adaptive response to harmful stress or malfunction.^33^ The physiological role of para-inflammation is to restore tissue functionality and homeostasis in disease events; however, it may become chronic inflammation if tissue stress or malfunction persists for a sustained period over several months and years.^33^ Para-inflammation has been identified as a potential role player in both the initiation and progression of the disease.^33^ In the retina, para-inflammation plays a protective homeostatic mechanism in the RGC layer and optic nerve head which have both shown to be affected in various HIV studies. Tezel^34^ suggests that increased stress over prolonged and cumulative periods fails the regulation of retinal immune response, leading to a neuroinflammatory degenerative process in the glial cells of the RGC layer and/or the retinal inner plexiform layer.^35^

Chronic oxidative stress prompts retinal para-inflammation leading to mitochondrial dysfunction and ultimately to retina dysfunction.^36^ Over time, oxidative damage causes mtDNA instability which leads to cumulative mitochondrial damage. This pathological process has been described in other ophthalmologic disorders such as diabetic retinopathy, age-related macula degeneration, and glaucoma,^37^ and therefore may also be related to the changes that are seen in HIV retinas.

Since the optic nerve is saturated with mitochondria, it is susceptible to impairment which can selectively damage RGCs.^38^ Axons within the optic nerve have a greater mitochondrial load. Underlying any potential loss of vision is the degeneration of the RGCs which form the optic nerve. Parameters to detect and quantify RGC damage are vital in the management of RGC-damaged neuropathies such as glaucoma and have been used in characterising the optic nerve head of HIV individuals on ART.^5,6,8,9,11,39,40^ Optical coherence tomography is the popular method of detecting such changes. However, these remain surrogate measures because they do not quantify the number of remaining or lost RGCs.^41^ The most commonly used parameter is the peripapillary retinal nerve fibre layer (ppRNFL) thickness.

Since RNFL is made up mostly of RGC axons, the measured thickness with OCT, it has a strong correlation with optic nerve axon count. The pattern of RGC damage indicates the type of cell that is affected. For instance, axonal damage affecting the magnocellular cells (M-cells), is reflected by RNFL thinning in the superior and inferior quadrants, whereas those that affect the parvocellular cells (P-cells) are reflected by the temporal thinning of the RNFL.^42^ The P-cell pattern is similar to what is described for mitochondrial optic neuropathies and is hallmarked by the temporal pallor of the optic disc.^42^ Various studies have reported that the temporal quadrant ppRNFL in PLWH is thinner than normal.^39,43,44^ Therefore, this may be a valid and novel indicator of the role of mitochondrial neuropathy, providing a potential disease mechanism for HIV-associated neuroretinal disorder.

Retinal pigment epithelial cells form a monolayer between the neuroretina and choroid. It has several vital functions, which include acting as an outer blood-retina barrier (oBRB), homeostasis of the neuroretina, and regulating the retinal immune response.^45^ Effects of ART, largely oxidative stress resulting in mitochondrial dysfunction, renders the blood-retina barrier (BRB) vulnerable to dysfunction which manifests as altered functioning and cellular senescence resulting in RPE ageing and age-related diseases similar to that seen in age-related macula degeneration (AMD).^37^ Since a similar pathogenesis is described in HIV, the functioning of the RPE must be explored.

### Mechanisms of HIV infection pathology

#### Tissue stress as a source of pathology

The retina has an immune system that is coordinated by immune cells such as the microglia, dendritic cells, and macrophages.^33^ Retinal microglia, RPE cells, together with choroidal macrophages/dendritic cells play a vital role in retinal homeostasis. In the ageing retina, they are the main contributors in regulating a stressed or malfunctioning retina and bring about retinal homeostasis. If this does not occur, the resultant inflammation causes endothelial dysfunction and microcirculatory abnormalities.^33^

Microvascular changes are believed to facilitate disease impairment to the neuroretina and therefore have an important role in exploring the pathogenesis of HIV retinal alterations. HIV infection is associated with increased inflammation-induced endothelial injury, endothelial activation, and endothelial dysfunction.^46^ HIV-related CVD is characterised by vascular endothelial activation and endothelial dysfunction. There is ample evidence about the correlation between retinal vascular changes and CVD.^47,48,49,50^

Retinal endothelial cells line the microvasculature that nourishes the neural retina.^48,51^ Hence, the inclusion of retinal vascular endothelial function assessment as a surrogate marker of CVD may prove valuable. The ophthalmic parameter that is an appropriate indicator to assess this factor is the ratio between the diameter of retinal arteries and retinal veins (A/V). The A/V ratio has been shown to be a fundamental ocular surrogate marker to reflect hypertension and atherosclerosis.^52^ A decreased A/V ratio is denoted by the narrowing of the arteries and widening of the veins thus indicating the risk of stroke and myocardial infarction ultimately making the retina susceptible to morphological changes in the retinal microvascular bed.

Pathai et al.^10^ explored vascular changes in HIV patients, and they discovered that arteriolar diameters narrowed with increasing duration of ART; independent of age; where those who were on 3 years of ART showed diameters of 167.83 mm while those who have taken ART for more than 6 years had diameters of 158.89 mm (*P* = 0.02). In a follow-up study Pathai et al.,^10^ investigating ART influences on retinal morphology, found that longer ART duration was associated with thinning of the inferior (*P* = 0.03) and nasal (*P* = 0.04) ppRNFL quadrants, and greater RNFL thickness of the superior quadrant with higher VL (HIV-negative individuals with 132.2 µm, and 133.8 µm in HIV-positive on ART with undetectable VL). Retinal nerve fibre layer thinning and retinal arteriolar narrowing may be associated with early vascular dysfunction in the nerve fibre layer (NFL).^10^ This hypothesis is supported by the location of large blood vessels in the retina. Large blood vessels are located in the superior and inferior quadrants and RNFL thinning was observed mainly in these areas in a previous study.^10^

Arcinue et al.^12^ reported an increased average total retinal thickness (*P* = 0.001) in the HIV-positive group at the fovea (232.6 µm ± 23.4 µm in HIV-positive patients and 213.1 µm ± 14.5 µm in HIV-negative patients). They reasoned that the retinal changes observed were likely due to retinal toxicity caused by ART used over long periods. Therefore, the association of detectable HIV viraemia and prolonged ART causing RNFL thickness changes is plausible and has been attributed to the process of para-inflammation.^33^ HIV viraemia may initiate a para-inflammatory process in the retina, which may manifest as increased thickness of the RNFL. Kalyani et al.^39^ propose that mitochondrial toxicity (caused by HIV or ART) may cause axonal damage to the RNFL, leading to an initial phase of swelling before atrophy, which is observed in some studies.^9,12,14^

**FIGURE 2:**
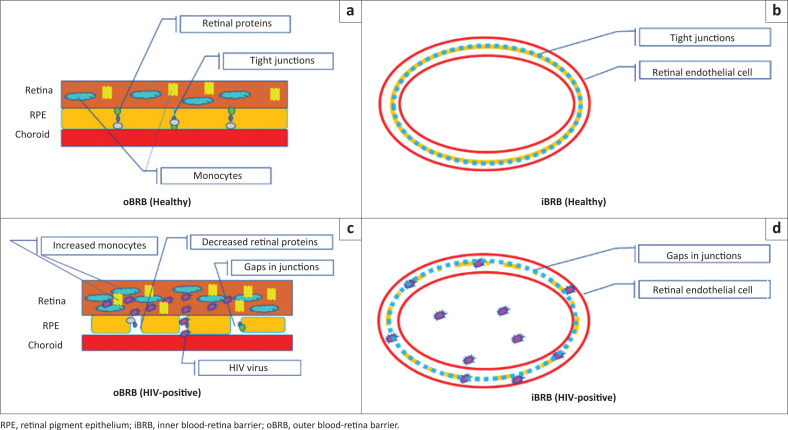
Proposed mechanism of retinal blood-retina barrier impairment in HIV.

While a close relationship between RNFL and the RPE is a prerequisite for normal vision,^53^ little is known about RPE involvement in PLWH retinas. A significant number of studies demonstrated that infectious agents cause damage to the BRB, specifically the oBRB.^48,54,55,56^ Tugizov^57^ showed that exposure to HIV increased the permeability of RPE monolayers due to the decreased expression of several vital proteins (ZO-1, occludin, and claudin), rather than altering cellular functions (Figure 2). These proteins are all involved in the maintenance of the BRB integrity and reduce RPE pathophysiology by stabilising the tight junctions. Disruption of these proteins caused BRB breakdown and resulted in infectious agent entry into the retina.^58^ The literature included in this review point to the source of retinal damage or altered function of the outer retina (RPE), which ultimately affects the inner retina (RNFL, outer plexiform layer [OPL], IPL, INL). The pathogenesis of the retina, particularly RPE, damage highlights a potentially important role of monocytes.

CD16+ monocytes (microglia and macrophages) play a role in autoimmune and chronic inflammatory diseases which contributes to neuroinflammation and neuronal damage which results in HIV-associated disorders in PLWH regardless of ART and immunocompetence.^59^ In the retina, microglia cells are located in the plexiform layers and function as immune surveillance of the retina.^38^ Research with retinal microglia has discovered, among others, CD4+ and CD16+ receptors; thus, suggesting that HIV infection of retinal microglia contributes to neural damage and RPE (considered the BRB) breakdown.^60^ HIV infection of RPE cells upregulates phagocytosis (destruction of foreign substances and removal of dead cells). When phagocytosis is disrupted, it leads to a build-up of ‘material’ in the retina and leads to an increased number of retinal macrophages within the macula which causes macula thickening that has been observed in PLWH who are immunocompetent.^60^ Similar findings were evident in other ocular conditions such as AMDs and diabetes. Clinical signs in diabetes and AMD, such as retinal hard exudation, oedema, and haemorrhages, indicate BRB impairment of varying severities.^61^ Research on retinal diseases (posterior uveitis, AMD, and diabetic retinopathy) has demonstrated BRB alterations at early disease stages promoted neuron injury.^61^ Therefore, the only mechanism by which circulating immune and inflammatory cells can enter the retina is through BRB impairment.

## The proposition of the blood-retina barrier as a gateway to neurosensory damage

The compounding effects of HIV-induced inflammation and oxidative stress from ART can have implications such as increased immune activation and increased endothelial dysfunction.^62^ Both HIV infection and ART can affect endothelial function, leading to impaired blood vessel health.^33,55^ HIV-induced inflammation and oxidative stress can damage endothelial cells, while some ART, particularly certain NRTIs, have been associated with mitochondrial toxicity and endothelial dysfunction. This combination can further compromise blood vessel integrity and contribute to vascular complications. The disarray of multiple risk factors culminates in the breakdown and failure of BRB performance, thereby initiating the events towards neurosensory damage. These findings suggest that the BRB is the mechanism of action or route of impairment due to the retinal inflammation in HIV. Consequently, it can be proposed that impairment of the BRB is either an early manifestation or a sign of advanced injury in retinal diseases. Regardless of the mechanism, the BRB is a pivotal point in HIV pathogenesis.^56,63^

The BRB is divided into an inner and outer portion. The inner blood-retina-barrier (iBRB) is formed by the inner capillary beds which lie in the ganglion nerve cell layer (Figure 2). The outer capillary bed lies in the inner and OPLs.^64^ The breakdown of the iBRB is followed by vascular damage and an increased risk of macula oedema.^65^ Three major constituents of the oBRB are the choroid, the Bruch’s membrane, and the RPE. The oBRB is the site where HIV-related inflammation originates; therefore, understanding the pathological processes that occur in the oBRB is vital for developing diagnostic or prognostic strategies.^66,67,68^

**FIGURE 3:**
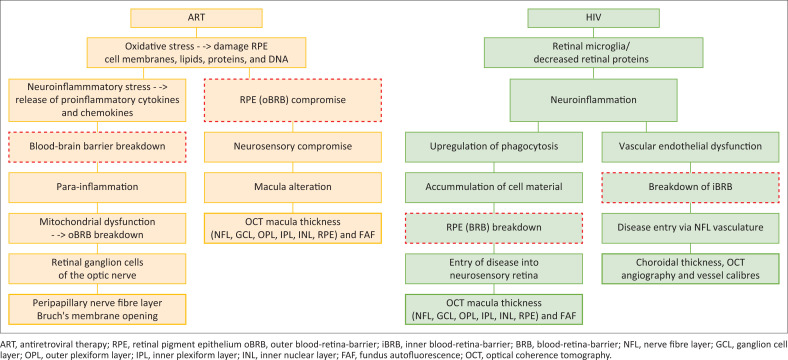
Clinical phenotype showing the blood-retina barrier as a gateway to neurosensory retinal HIV alterations.

It may be possible to develop a clinical phenotype as shown in Figure 3 based on the onset of the disease, disease severity, and drug intake. Being able to link clinical phenotypes to known disease mechanisms will aid in determining the potential targets necessary for diagnosis and management.

In HIV infection, immune activation and inflammation lead to disruption of the BRB. Infected immune cells and pro-inflammatory molecules can compromise the integrity of the RPE tight junctions and retinal vascular endothelial cell junctions, resulting in increased permeability of the BRB. This disruption allows HIV particles and infected immune cells to permeate the BRB and affect the retinal tissue (neuronal dysfunction, and loss of RGCs). The release of pro-inflammatory cytokines, chemokines, and immune mediators in the retinal microenvironment can lead to neurotoxic effects and cellular damage. This cascade of events ultimately manifests as the clinical phenotype of HIV/ART. The clinical phenotype of HIV/ART reflects the effects of BRB disruption and subsequent neurosensory retinal alterations. Diagnostic techniques such as OCT imaging can reveal macula oedema, changes in inner retinal layers, and integrity of the outer segment. Optical coherence tomography plays a pivotal role in assessing the integrity of the BRB and evaluating neurosensory retinal alterations.

## The role of advanced imaging in detecting subclinical blood-retina barrier changes in people living with HIV

Imaging technological advances have transformed the assessment of the BRB and substantially increased our understanding of retinal diseases. There is limited evidence on how to assess the RPE to support the hypothesis of a BRB compromise on HIV and long-term ART. However, there are ophthalmic procedures that can quantitively or qualitatively assess the integrity of the RPE. Among the most useful techniques in clinical practice and research are fluorescein angiography (FA), indocyanine green angiography, OCT, and fundus autofluorescence.^69^ These imaging modalities lead to improved patient outcomes and the development of targeted therapeutics for different retinal illnesses by accurately identifying and monitoring the integrity and function of the BRB.

The most extensively used imaging technique for assessing the BRB is FA.^70^ A fluorescent dye (fluorescein) is injected into a vein during this treatment, and as it circulates through the bloodstream, a series of photos are taken to see the dye’s journey through retinal blood vessels. This aids in detecting anomalies in retinal blood flow and leaks in the BRB, both of which are signs of BRB failure. However, FA is only semiquantitative and its reproducibility depends on the inconstant quality of the angiograms.^70^ Similar to FA, indocyanine green angiography employs an indocyanine green dye. This technique permits the viewing of deeper choroidal veins. Indocyanine green angiography complements FA by emphasising choroidal circulation and gives a more comprehensive assessment of the BRB. The major drawback of the techniques that have been mentioned is their invasiveness, therefore placing OCT and OCT angiography (OCTA) as superior options for evaluation, diagnosis, and management.

While OCT has been used widely in clinical practice, there is still some uncertainty concerning its role in disease diagnosis and management. Optical coherence tomography can provide retinal features that can act as retinal quantitative imaging biomarkers to refer to objective and quantitative measures of the retina that can be obtained using various imaging techniques.^70^ These biomarkers can be used to detect and monitor retinal changes associated with various diseases and their treatment modalities, including HIV infection and ART medications. Most research publications reviewed in this study point to the strong associations of HIV and ART with the retina when measuring the thickness of the central macula, NFL, ganglion cell layer (GCL), INL, ganglion cell and inner plexiform layer (GCIPL), ganglion cell complex (GCC), and ppRNFL.^5,6,9,12,13,14,43,44,71^

The use of OCTA is a novel method that allows non-invasive three-dimensional viewing of the microvasculature within the eye at high resolution. It utilises light waves to detect the movement of red blood cells within the ocular vessels. Most studies that have evaluated PLWH found that in patients with longstanding HIV and ART use, OCTA found microvascular abnormalities (decrease in the density of the capillary network) even among eyes without clinical retinal pathology.^72,73^ This means that retinal microvasculature may be a valuable ‘preclinical biomarker for disease processes’ in PLWH.^73^

When evaluating the RPE, evidence shows that OCT data from areas of disrupted oBRB differ significantly from OCT data from areas of intact oBRB.^69^ Data from OCT histograms have shown a consistent difference in their distributions when comparing one area of OCT from the intact oBRB region to the same area in the case of a disrupted oBRB area.^69^ Some OCT technologies have an in-built fundus autofluorescence (FAF) technology which provides a non-invasive mapping of changes at the level of the RPE-photoreceptor complex as well as changes in macula pigment distribution. Fundus autofluorescence imaging measures the natural fluorescence (lipofuscin) emitted by RPE. Excessive lipofuscin accumulates in RPE cells when there is impaired barrier function. Only states of immunocompromise in HIV with the presence of cytomegalovirus retinitis (CMVR) have been evaluated with FAF. These studies all found hyperfluorescence, indicating a lipofuscin build-up in the RPE.^74,75,76^ Future studies of PLWH without CMVR could allude to the possible activity at the level of RPE in which the HIV is affecting the tissues which ultimately leads to tissue destruction.

Early research observations suggest that patients with HIV may develop impaired choroidal perfusion, a pathogenesis which is linked to vascular endothelial dysfunction attributed to direct toxic effects of HIV.^77^ In recent years, the measurement of choroidal thickness with OCT has been described as an imaging biomarker to identify subclinical inflammation in various diseases, especially for conditions with a vascular component. The choroid of patients thickens in active phases of inflammatory diseases with vascular involvement. There are only two studies on HIV patients which were found for this review.^13,72^ These studies agree that the choroid is thicker in HIV patients as compared to healthy controls.^72^ However, it is thinner in newly diagnosed cases and becomes thicker with long-term ART.^13^ The results support the view that HIV or ART inflammation could lead to increased vascular permeability and endothelial cell activation. This suggests that choroidal thickness may be a biomarker for the effects of ART treatment on the iBRB.

## Future directions for clinical research on HIV and the retina

### Ground truth for prognostic prediction models in HIV-related retinal para-inflammation

One of the challenges in characterising HIV/ART-related changes is the lack of information on normative values for OCT parameters, or quantifiable ‘ground truth’. The novel biomarkers of inflammation and neurodegeneration in the retina have the potential to increase our knowledge of the systemic inflammatory effects, and thus provide a non-invasive method to monitor the HIV/ART status and facilitate multidisciplinary interaction when dealing with patients.

To improve patient outcomes and guide clinical decision-making, prognostic prediction models have gained prominence in assessing disease progression and prognosis. However, the development of accurate and reliable prognostic models hinges on the availability and utilisation of ground truth data. In the context of prognostic prediction models for HIV-related retinal para-inflammation, ground truth encompasses comprehensive and validated clinical data collected from patients over time. Clinical prediction models require more predictors from patients’ clinical findings, history, or investigation results. These data should include detailed information about disease characteristics, progression, treatment regimens, and outcomes.

We propose the development of an ‘HIV/ART Retinal Para-Inflammation Prognostic Score’ (HARPP score) to predict the risk of disease progression and severity in PLWH on ART. Based on age, duration of HIV infection, CD4 cell count, VL, and use of ART, patient outcomes (morphological changes causing visual function changes) can be predicted through clinical prediction rules. It may provide a quantitative score that represents their estimated risk of disease progression. The HARPP score should be designed to assess and predict the risk of retinal complications associated with chronic immune activation and inflammation in PLWH based on the evaluation of several clinical and imaging parameters that reflect the degree of retinal inflammation and associated changes. These parameters may include clinical features such as retinal vasculitis, retinal haemorrhages, cotton wool spots, and macula oedema, as well as OCT imaging findings such as retinal thickness of the RNFL, GCIPL, RPE, and choroid. By assigning a score based on the presence and severity of these parameters, the HARPP score will stratify the level of retinal para-inflammation in PLWH on ART and predict the risk of retinal complications. This score can help clinicians in risk stratification and treatment planning. Patients with higher HARPP scores may be considered at greater risk of severe disease and could be recommended for more frequent ophthalmologic evaluations, earlier initiation of treatment, or closer monitoring. Future clinical trials are required to validate the efficacy and applicability of the novel biomarkers across diverse cohorts. Rigorous validation studies will ensure the robustness and generalisability of HARPP, ultimately facilitating its integration into clinical practice for more accurate prognostic evaluations in PLWH on ART.

## Conclusion

The cutting-edge OCT-based layer segmentation has allowed for the evaluation of the progression of neurodegeneration in the retina on a structural level. While significant progress has been made in understanding the impact of HIV and ART on the retina, there are still many unknowns, and further research is needed to fully understand the complex relationship between HIV, ART, retinal, and visual health and the role of inflammation in this population. Nonetheless, the evidence gleaned from this literature search indicates that although it may be a challenge to differentiate inflammation caused by HIV versus that of ART, the cascade of damage is initiated in the BRB. Therefore, future research should be focused on the preservation of the BRB in efforts to minimise or rehabilitate the effects of HIV and long-term ART.
